# Outlines of Surgical Diagnosis

**Published:** 1864-10

**Authors:** 


Outlines of Surgical Diagnosis. By George H. B. Macleod, M.D., F.R. C.S.E.; Fellow of Faculty of Physicians and Surgeons of Glasgow; Lecturer on Surgery at Andersonia's University, &c., &c., &c. First American Edition, reprinted from advanced sheets. New York: Bailliere Brothers, No. 520 Broadway. 1864.

This is a neatly published octavo volume of 505 pages. In the introductory chapter, of sixty pages, the author briefly discusses the conditions, means, and methods of diagnosis. In the remainder of the work, he gives a concise statement of the important points of diagnosis in tumors, morbid growths, fractures, dislocations, and surgical diseases generally. From such examination as we have been able to make, we think the student, and young surgeon especially, will find the work a valuable one for study and reference.

